# Improving plant productivity by re‐tuning the regeneration of RuBP in the Calvin–Benson–Bassham cycle

**DOI:** 10.1111/nph.18394

**Published:** 2022-08-07

**Authors:** Christine A. Raines

**Affiliations:** ^1^ School of Life Sciences University of Essex Wivenhoe Park Colchester Essex CO3 4JE UK

**Keywords:** biotechnology, Calvin–Benson–Bassham Cycle, modelling, multigene, photosynthesis, transgenic

## Abstract

The Calvin–Benson–Bassham (CBB) cycle is arguably the most important pathway on earth, capturing CO_2_ from the atmosphere and converting it into organic molecules, providing the basis for life on our planet. This cycle has been intensively studied over the 50 yr since it was elucidated, and it is highly conserved across nature, from cyanobacteria to the largest of our land plants. Eight out of the 11 enzymes in this cycle catalyse the regeneration of ribulose‐1‐5 bisphosphate (RuBP), the CO_2_ acceptor molecule. The potential to manipulate RuBP regeneration to improve photosynthesis has been demonstrated in a number of plant species, and the development of new technologies, such as omics and synthetic biology provides exciting future opportunities to improve photosynthesis and increase crop yields.

**Table:** 

	Contents	
	Summary	350
I.	Introduction	350
II.	Transgenic manipulation of RuBP regeneration	352
III.	Modelling to take a more predictive approach to identify targets to improve the Calvin–Benson–Bassham cycle?	353
IV.	Gaps in our knowledge about the Calvin–Benson–Bassham cycle	354
V.	Conclusions	354
	Acknowledgements	355
	References	355

## I. Introduction

**Fig. 1:**
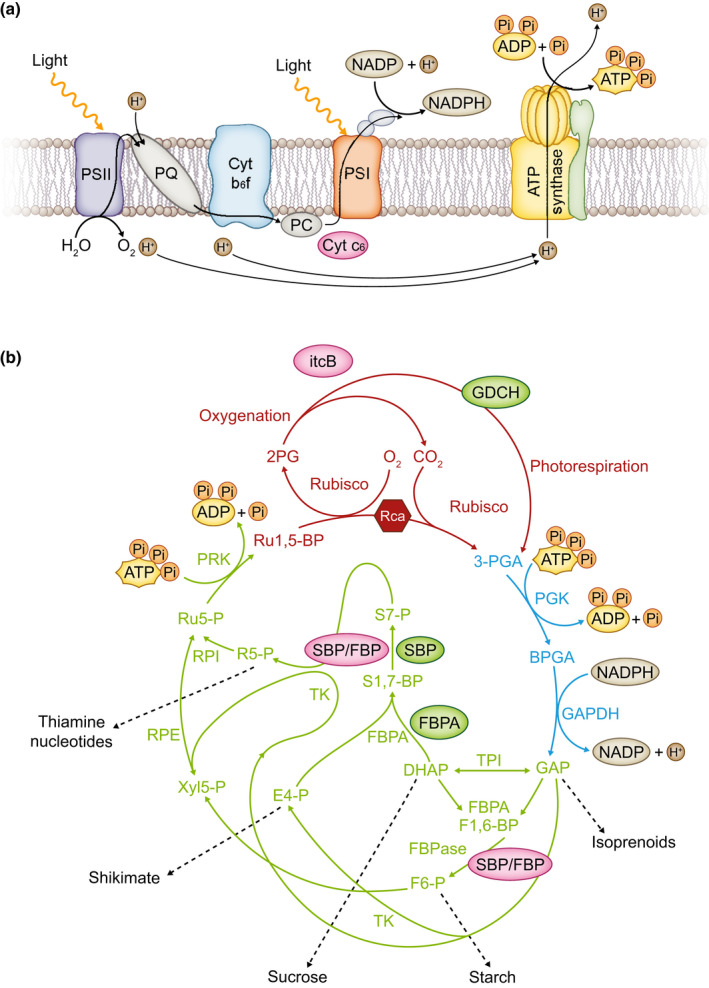
The Calvin–Benson–Bassham (CBB) cycle. (a) Energy in the form of ATP and NADPH needed to drive the CBB cycle is produced in the thylakoid membrane‐located electron transport chain. (b) The first step in the CBB cycle is catalysed by ribulose 1,5‐bisphosphate carboxylase/oxygenase (Rubisco) resulting in the formation of 3‐phosphoglycerate (3‐PGA). The next two reactions form the reductive phase and are catalysed by phosphoglycerate kinase (PGK), forming glycerate 1,3‐bisphosphate (BPGA) using ATP and glyceraldehyde 3‐phosphate dehydrogenase (GAPDH) which forms glyceraldehyde 3‐phosphate (GAP) consuming NADPH. Triose phosphate isomerase (TPI) catalyses the production of dihydroxyacetone phosphate (DHAP) and together with GAP enters the regenerative phase of the cycle, catalysed by fructose 1,6‐bisphosphate/sedoheptulose 1,7‐bisphosphate aldolase (FBPA), forming sedoheptulose 1,7‐bisphosphate (S1,7‐BP) and fructose 1,6‐bisphosphate (F1,6‐BP). Sedoheptulose 1,7‐bisphosphatase (SBPase) and FBPase (fructose 1,6‐bisphosphatase) then produce sedoheptulose 7‐phosphate (S7‐P) and fructose 6‐phosphate (F6‐P), which are converted to 5C compounds in reactions catalysed by transketolase (TK), ribose 5‐P isomerase (RPI) and ribulose 5‐phosphate epimerase (RPE), resulting in the formation of ribulose 5‐P (Ru5P). The final step in the cycle is catalysed by ribulose 5‐phosphate kinase (PRK), producing the CO_2_ acceptor molecule ribulose 1,5‐bisphosphate (Ru1,5‐BP). The three phases of the CBB cycle are shown: (1) carboxylation (red), (2) reduction (blue) and (3) regeneration (green). The products of the CBB cycle are exported to a number of biosynthetic pathways (grey dashed lines): isoprenoid, starch, sucrose, shikimate, thiamine and nucleotide. Rubisco has a competing oxygenase reaction which results in the formation of 2‐phosphoglycerate, which enters the photorespiratory pathway. The manipulations related to ribulose‐1‐5 bisphosphate (RuBP) regeneration discussed in this paper are in the electron transport chain algal cytochrome C6 (CytC6), the photorespiratory cycle H‐subunit of glycine decarboxylate (GDCH), the putative transporter from an alga (ictB), the endogenous SBPase and FBPA enzymes and the cyanobacterial bifunctional sedoheptulose 1,7‐bisphosphatase/fructose 1,6‐bisphosphatase (SBPase/FBPase) enzyme. Overexpression of endogenous proteins is shown in green and foreign proteins in pink.

The Calvin–Benson–Bassham (CBB) cycle is the primary photosynthetic pathway for assimilation of atmospheric CO_2_ in over 85% of terrestrial plants, which are named C3 species as the first stable product of this cycle is a three‐carbon compound, glycerate3‐phosphate (Geiger & Servaites, 1994; Sharkey, 2019). The CBB cycle involves 11 enzymes, and the biochemical steps have been divided into three stages: carboxylation carried out by 1,5‐bisphosphate carboxylase/oxygenase (Rubisco), reduction, and RuBP regeneration (Fig. 1). Under light saturating and CO_2_‐limiting conditions Rubisco activity is the major determinant of the efficiency of carbon fixation, but as CO_2_ levels rise and light intensity decreases, this balance shifts towards both the reductive and regenerative phases of the CBB cycle that catalyse the synthesis of the CO_2_ acceptor molecule, RuBP (Fig. 1). Improving photosynthesis has been identified as a target to increase crop yield based on theory, modeling and empirical studies (Box 1). A major focus of efforts to improve photosynthesis is still the enzyme Rubisco, through the application of protein engineering strategies and also via manipulation of its expression in transgenic plants (Parry *et al*., 2012; Zhou & Whitney, 2019; Yoon *et al*., 2020; Makino, 2021). However, manipulating the expression of other enzymes of the CBB cycle can also enhance photosynthesis and growth. The aim of this insight will be to highlight the current status and future potential to improve the processes leading to regeneration of RuBP to deliver a step change in photosynthesis and boost crop yield.

Box 1 **Why target photosynthesis to increase crop yields?**

There is a pressing need to increase the yield of crop plants in order to feed our growing population, and this needs to be achieved within the next 20 yr without increasing land area used, or increasing water or nutrient inputs. The yield potential is the maximum yield attainable from a crop when the best adapted crop variety is grown, in optimal conditions with no biotic or abiotic stress. Yield potential is determined by a combination of the availability of light, the ability to capture the available light, conversion of the fixed energy into biomass, and plant architecture in terms of harvest index. Energy conversion is the only one of these four components that is well below its potential maximum, and this parameter is determined by photosynthetic efficiency (Zhu *et al*., 2010). The significance of photosynthetic efficiency for yield can be described by the following equation:

$$W_{h}=S\times e_{i}\times e_{c}\times e_{p}$$

*W*_h_, harvested yield; *S*, solar energy; *e*_i_, light interception efficiency; *e*_c_, energy conversion efficiency; *e*_p_, harvest index.

**Fig. 2:**
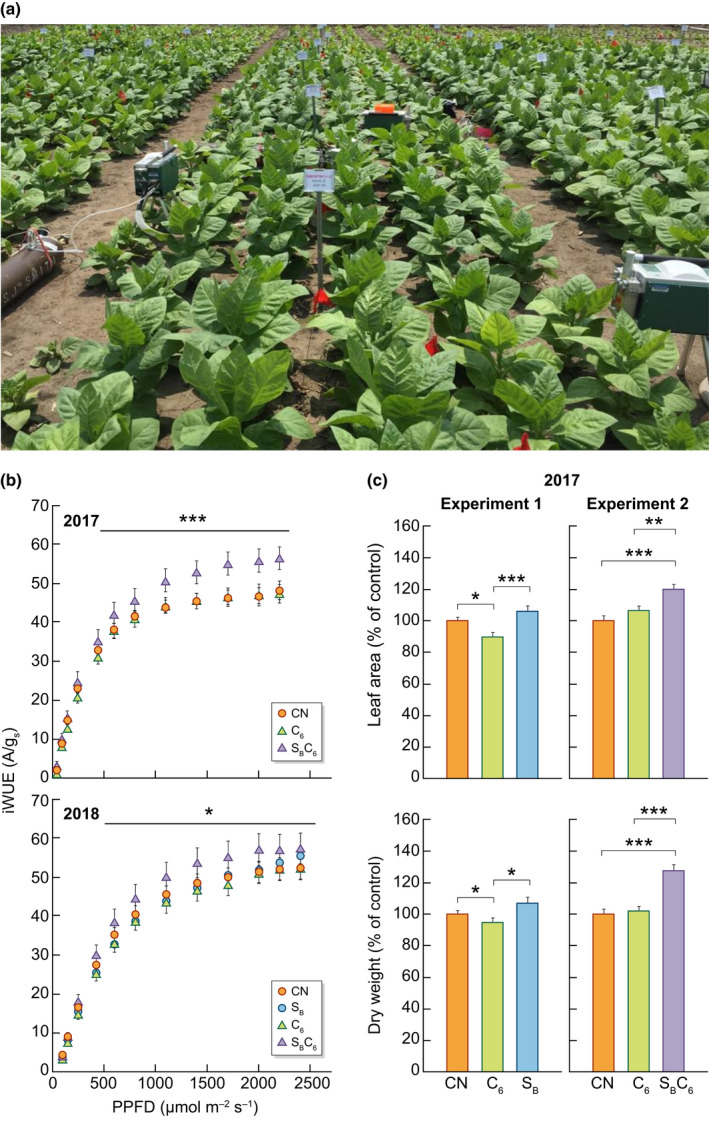
Expression of sedoheptulose 1,7‐bisphosphatase/fructose 1,6‐bisphosphatase and cytochrome C6 in transgenic tobacco improves water use efficiency and biomass in field‐grown plants. (a) Transgenic tobacco plants in field plots in Illinois. (b) Intrinsic water use efficiency and (c) biomass are increased in field‐grown transgenic plants expressing the bifunctional sedoheptulose 1,7‐bisphosphatase/fructose 1,6‐bisphosphatase (SBPase/FBPase) enzyme together with the algal cytochrome C6. CN, wild‐type and azygous controls; A/gs, assimilation rate over stomatal conductance; C, cytochrome C6; iWUE, intrinsic water use efficiency; PPFD, photosynthetic photon flux density; S_B_, SBPase/FBPase (Lopez‐Calcagno *et al*., 2020). In (b), asterisks indicate significant differences between the transgenic plants and the controls, using a linear mixed‐effects model and type III analysis of variance (ANOVA) and contrast analysis (*, *P* < 0.05; **, *P* < 0.01; ***, *P* < 0.001); in (c), asterisks indicate significance between transgenic plants and controls, or between genotypes, using ANOVA with Tukey's post‐hoc test (*, *P* < 0.05; **, *P* < 0.01, ***, *P* < 0.001). This figure is modified from figures presented in Lopez‐Calcagno *et al*. (2020).

Although there have been some doubts expressed about the strategy of targeting photosynthesis to improve yield, evidence that increased yield can be obtained by improving photosynthetic CO_2_ assimilation (the Calvin–Benson–Bassham (CBB) cycle) comes from studies with a range of plants grown in field conditions under elevated CO_2_ (Ainsworth & Long, 2021). Further evidence has come from transgenic manipulation of photosynthesis that showed under field conditions that an increase in photosynthesis and biomass was obtained (South *et al*., 2019). An increase in grain yield in rice grown in paddy fields was also found in plants with increased levels of ribulose 1,5‐bisphosphate carboxylase/oxygenase (Rubisco) (Yoon *et al*., 2020), and increased biomass and water use efficiency was observed in field‐grown tobacco plants expressing the cyanobacterial sedoheptulose 1,7‐bisphosphatase/fructose 1,6‐bisphosphatase (SBPase/FBPase) and the algal cytochrome C6 (see Fig. 2).

## II. Transgenic manipulation of RuBP regeneration

Synthesis of the CO_2_ acceptor molecule, RuBP, requires the two steps in the reductive phase of the cycle to produce the C3 molecule glyceraldehyde 3‐phosphate (GAP) utilizing ATP and NADPH from the light reactions. The biochemical steps in the regenerative phase of the cycle use this GAP, and through the action of eight enzymes catalysing 10 reactions produce RuBP. In the 1990s antisense technology was used as an empirical approach to identify which of these enzymes exert control over the flow of CO_2_ in this pathway (Fig. 1). These studies demonstrated that no one enzyme had complete control over CO_2_ assimilation under all environmental conditions and, over and above Rubisco, identified sedoheptulose 1,7‐bisphosphatase (SBPase), fructose 1,6‐bisphosphate aldolase (FBPA) and transketolase (TK) as promising targets for overexpression and improvement of photosynthesis (Stitt & Schulze, 1994; Raines, 2003). Based on these empirical studies, a transgenic overexpression approach has shown that increasing the levels of SBPase can improve photosynthesis and growth in algae and a number of plant species including tobacco (in the field and glasshouse), wheat, rice, and Arabidopsis (Lefebvre *et al*., 2005; Simkin *et al*., 2015; Driever *et al*., 2017; Suzuki *et al*., 2019). Furthermore, tomato plants with increased SBPase activity were found to be more chilling tolerant with increased photosynthetic capacity (Ding *et al*., 2017). Overexpression of FBPA in tobacco also resulted in positive effects on photosynthesis and biomass (Uematsu *et al*., 2012; Simkin *et al*., 2015), and in tomato an increase in seed weight under both optimal and sub‐optimal temperatures was observed (Cai *et al*., 2022). Introduction of the bifunctional cyanobacterial CBB cycle enzyme sedoheptulose 1,7‐bisphosphatase/fructose 1,6‐bisphosphatase (SBPase/FBPase) into tobacco plants, lettuce and soybean (under elevated CO_2_), has also resulted in improved CO_2_ assimilation and growth (Miyagawa *et al*., 2001; Ichikawa *et al*., 2010; Kohler *et al*., 2017; Benes *et al*., 2020).

Given the central role of the CBB cycle in primary carbon metabolism, improvements in RuBP regeneration can also be realized through the combined introduction of proteins that function outside of the CBB cycle (Fig. 1). Two examples of this approach are as follows: the putative transporter ictB when introduced into tobacco in combination with SBPase and FBPA resulted in a further improvement in photosynthesis and growth over single gene manipulations, and overexpression of the H subunit of glycine decarboxylase together with SBPase and FBPA in Arabidopsis also resulted in additional positive effects when compared to the single manipulations (Simkin *et al*., 2015; Simkin *et al*., 2017). RuBP regeneration can also be limited by the availability of the ATP and NADPH produced by light reactions. To remove this potential bottleneck, plants were produced with a combination of overexpression of either the endogenous SBPase enzyme or bifunctional cyanobacterial SBPase/FBPase together with the algal cytochrome C6 protein, which rapidly transfers electrons from the cytochrome b6/f complex to photosystem I. Interestingly, tobacco plants carrying these manipulations were shown to exhibit not only improved photosynthesis and yield but also improved water use efficiency when grown in field conditions (Fig. 2) (Lopez‐Calcagno *et al*., 2020). Another more recent example is a study in which the co‐overexpression of SBPase and cytosolic FBPase showed a synergistic effect in transgenic tobacco plants, resulting in improvements in biomass, plant height, stem diameter and pod weight (Li *et al*., 2022). Additional combinations of targets for improving RuBP regeneration have been proposed. For example, overexpression of triose phosphate isomerase (TPI) in conjunction with other CBB cycle enzymes may provide further enhancements in carbon assimilation, by removing triose phosphate limitation (Suzuki *et al*., 2022). The expression of a group of CBB cycle genes (*FBA1*, *RCA1*, *FBP5* and *PGK1*) was increased when the expression of the Brassinole resistant 1 transcription factor (BZR1) was increased and enhanced photosynthetic capacity was observed, suggesting that simultaneous overexpression of these proteins may stimulate the CBB cycle (Yin *et al*., 2022). These new findings, together with advancements in modeling, open up opportunities to re‐engineer the CBB cycle to maximize improvements.

## III. Modelling to take a more predictive approach to identify targets to improve the Calvin–Benson–Bassham cycle?

A number of models incorporating the CBB cycle have been established, and the major strength of these mathematical models is that they offer a tool with which to study a range of scenarios and simulations that would not be possible experimentally. Kinetic models of the CBB cycle raised the hypothesis that SBPase was a major control point in the CBB cycle and that Rubisco is not always the sole limiting factor (Janasch *et al*., 2018). A model using an evolutionary algorithm suggested that simultaneous overexpression of SBP and FBPA could lead to significant improvement in CO_2_ assimilation rates (Zhu *et al*., 2007). Experimental evidence supporting this hypothesis came from a study showing that the combined over‐expression of SBPase and FBPA in tobacco resulted in a cumulative increase in biomass (Simkin *et al*., 2015).

A recent modelling study has hypothesised that sub‐cycles may exist within the CBB cycle and proposed that excessive increases in the activity of individual enzymes in one sub‐cycle could impact on the availability of metabolites for other sub‐cycles. The impact of this metabolite imbalance would be to lower reaction rates and the overall CBB cycle flux (Zhao *et al*., 2020). Interestingly, increasing the TK levels in tobacco resulted in a negative growth phenotype, reduced rates of photosynthesis and partial thiamine auxotrophy, suggesting that this manipulation of the CBB cycle had caused an imbalance in flux out of the cycle (Khozaei *et al*., 2015).

A model of the CBB cycle that included the electron transport chain has revealed the importance of balancing supply and demand reactions in order to ensure the efficiency of the CBB cycle. This merged model raised the hypothesis that a ‘standby’ mode during light–dark transitions may be essential to allow the CBB cycle to restart under increasing light, and a role for the oxidative pentose phosphate pathway was proposed (Matuszynska *et al*., 2019). The outputs from this model are supported by experimental studies showing that the CBB cycle relies on carbon influx from anaplerotic reactions to compensate for the depletion of intermediates, particularly under the fluctuating light conditions found in natural environments (Makowka *et al*., 2020; Xu *et al*., 2022).

## IV. Gaps in our knowledge about the Calvin–Benson–Bassham cycle

Although the CBB cycle is ubiquitous and highly conserved between species, there remains a number of significant gaps in our knowledge, including the following.

### The extent of natural variation that exists in the kinetic parameters and regulation of individual enzymes is unknown

Individual CBB cycle enzymes from different C3 species can exhibit diversity in their primary protein sequences but, with the exception of Rubisco, the functional implication of this diversity has not been studied systematically, and to date detailed data on the catalytic properties of any individual enzyme of the cycle is available for only a very few species. Extending our knowledge of the natural diversity of these enzymes will allow a better understanding of the relationship between the structure/function of the CBB cycle enzymes and the specific catalytic roles of the conserved and nonconserved amino acids. To achieve this the catalytic diversity needs to be analysed alongside the CBB cycle enzyme sequences, to identify potential catalytic switches for improving photosynthesis and productivity. Four enzymes involved in the CBB cycle, GAPDH, SBPase, FBPase and PRK, are regulated by light via redox changes in the chloroplast, through the thioredoxin (trx) system and the CP12 protein. Increasing the expression of these regulators in conjunction with their CBB cycle targets has yet to be explored as a strategy but could be a viable option given the indications from overexpression of trx on its own (Nikkanen & Rintamäki, 2019; Gurrieri *et al*., 2021).

### Genetic factors regulating the coordinated expression of the C3 cycle gene are not known for even one species

The RNA abundance of CBB cycle enzymes changes during plant development, in response to light conditions and the accumulation of sugars; however, the detailed molecular mechanisms underlying the regulation of individual genes have not been elucidated, and even less is known about the coordination of expression of the whole pathway (Wang *et al*., 2017). The availability of whole genome sequences, including those of crop plants, together with the advent of omics technologies, eQTL and bioinformatics advances has enabled new approaches. A study of *Populus tomentosa* identified 40 transcription factors with potential roles in the regulation of 46 CBB cycle genes (Wang *et al*., 2019). Ten of these transcription factors were explored in more detail using a combination of metabolic analysis and outputs from gene regulatory network analysis. Interestingly, half of the SNPs identified were located in the promoter regions of the genes. Promoter scanning results revealed that 121 *cis*‐motifs co‐occurred in 80% of promoters of genes involved in the CBB cycle. The value of this approach is that it can provide insight into common regulatory mechanisms that would enable multitarget nontransgenic (gene editing) approaches to be incorporated into strategies to improve RuBP regeneration and CO_2_ fixation.

### The regulation of the allocation of carbon through and from the C3 cycle to adjacent pathways has not been addressed holistically

Metabolite profiling of CBB cycle intermediates from C3 and C4 species revealed specificity and diversity in the CBB cycle between C3 species (Stitt *et al*., 2021). A comparative study between Arabidopsis and rice showed that these two C3 species prioritise different reactions when exposed to changes in irradiance (Borghi *et al*., 2019). The implication of these findings is that strategies to improve photosynthesis will need to be tailored depending on the crop, highlighting the need for systematic analyses of target species and cultivars within the same species. It is unlikely that metabolic profiling could be used to screen as a high‐throughput, automated approach. This type of study is likely to be most beneficial to target individual species in different environments or to provide data that can be built into models.

## V. Conclusions

Advances in kinetic flux and multiscale modelling have provided novel predictions on how to further enhance RuBP regeneration. Testing these outputs will require the application of rapid high‐throughput and iterative approaches to identify the best candidates with which to achieve improvements in photosynthesis (Benes *et al*., 2020). At the same time, new approaches enabling identification of genetic factors and mechanisms involved in regulating the expression of CBB cycle genes will underpin the application of gene‐editing technologies to modify this pathway. Excitingly, it may even be possible to use synthetic biology to build a completely synthetic, more efficient CO_2_ fixation pathway to operate in parallel with the endogenous cycle (Erb & Zarzycki, 2016; Schwander *et al*., 2016; Löwe & Kremling, 2021) or to introduce improved enzymes to operate within the existing cycle. The advent of these new technologies provides future researchers with an exciting toolbox with which to exploit the full potential that improvements in RuBP regeneration can contribute to increasing photosynthetic performance and crop yield.
